# Real-Time Markerless Tooth Detection Towards Dynamic Robot-Assisted Dental Implant Navigation

**DOI:** 10.3390/dj14060345

**Published:** 2026-06-05

**Authors:** Vasile Bulbucan, Daria Pisla, Paul Tucan, Cristian Dinu, Calin Vaida, Rares Mocan, Mihaela Baciut, Sebastian Stoia, Mihaela Hedesiu, Ionut Zima, Doina Pisla, TEAM Project Group

**Affiliations:** 1 CESTER, (Research Center for Industrial Robots Simulation and Testing), Department of Mechanical Systems Engineering, Faculty of Industrial Engineering, Robotics and Production Management, Technical University of Cluj-Napoca, 28 Memorandumului Street, 400114 Cluj-Napoca, Romania; vasile.bulbucan@mep.utcluj.ro (V.B.); paul.tucan@mep.utcluj.ro (P.T.); calin.vaida@mep.utcluj.ro (C.V.); ionut.zima@mep.utcluj.ro (I.Z.); doina.pisla@mep.utcluj.ro (D.P.); 2 European University of Technology, European Union; 3 Department of Maxillofacial Surgery and Radiology, Oral Radiology, “Iuliu Hatieganu” University of Medicine and Pharmacy, 400006 Cluj-Napoca, Romania; rares.cris.mocan@elearn.umfcluj.ro (R.M.); mhedesiu@umfcluj.ro (M.H.); 4 Department of Maxillofacial Surgery and Radiology, Maxillofacial Surgery and Implantology, “Iuliu Hatieganu” University of Medicine and Pharmacy, 400429 Cluj-Napoca, Romania; mbaciut@umfcluj.ro (M.B.); sebastian.stoia@umfcluj.ro (S.S.); 5 Technical Sciences Academy of Romania, B-dul Dacia, 26, 030167 Bucharest, Romania

**Keywords:** artificial intelligence, robot-assisted oral surgery, segmentation, dental imaging, YOLO, dental implants

## Abstract

**Background/Objectives:** Dynamic navigation and robot-assisted implant workflows depend on robust intraoral perception. Marker-based tracking introduces workflow complexity and is sensitive to occlusions, motivating markerless alternatives. This study evaluates whether a single-stage YOLO instance segmentation model (YOLO-seg) can provide a practical markerless perception layer for dental navigation, combining accurate per-tooth delineation with low, predictable inference latency. **Methods:** YOLO-seg was trained end to end on an intraoral RGB corpus of 400 training, 20 validation, and 100 testing images, combining a public source and a partner-hospital in-house set. A two-stage YOLO + SAM baseline was implemented for comparison. Segmentation quality was evaluated on a 50-image held-out clinical test set at three complementary levels (per-instance matching, per-class union, and global union), with paired Wilcoxon signed-rank tests, Cliff’s delta effect sizes, and 95% bootstrap confidence intervals. Runtime was assessed under matched inference-only and end-to-end conditions on *N* = 100 frames at a 640 × 640 resolution on an NVIDIA RTX A2000 GPU. **Results:** YOLO-seg significantly outperformed YOLO + SAM across all primary metrics, with very large effect sizes (Cliff’s delta: 0.76–0.94; Wilcoxon *p* < 10^−8^ on every metric except precision at IoU ≥ 0.5). YOLO-seg reached AP_50_ = 0.716 and recall = 0.973 versus 0.383 and 0.398 for YOLO + SAM. Under matched inference-only timing, YOLO-seg ran at 27.08 ms per frame (36.9 FPS) versus 1302.78 ms (0.77 FPS), an approximately 48-fold latency gap intrinsic to the two-stage forward pass. **Conclusions:** YOLO-seg shows strong potential as a 2D perception module for dental navigation, balancing per-instance segmentation fidelity with real-time feasibility under the tested conditions. These results support its use as a 2D perception front-end for future integration with stereo-based 3D reconstruction and robot-assisted navigation; 3D registration accuracy, implant-placement error, and robotic execution remain outside the scope of the present study.

## 1. Introduction

Accurate dental implant placement requires high spatial precision due to the proximity of critical anatomical structures such as the inferior alveolar nerve and the maxillary sinus. Even small deviations between the planned and executed implant trajectory may lead to clinically significant complications, motivating the increasing adoption of computer-assisted implant surgery systems [1,2]. There are two main approaches regarding dental implant placement, namely static surgical guides (patient-specific drill templates for implant positioning) and dynamic navigation solutions, and both have demonstrated improved accuracy compared to freehand techniques, with dynamic approaches offering increased intraoperative flexibility [3]. Recent meta-analyses confirm a consistent accuracy hierarchy with robot-assisted and computer-assisted methods, outperforming freehand placement across coronal, apical, and angular deviation metrics [4]. Most commercially available dynamic navigation systems rely on optical tracking with external fiducial markers or marker holders attached to the patient or surgical instruments. While these systems can achieve high accuracy under controlled conditions, they introduce additional calibration steps and workflow complexity and remain sensitive to line-of-sight interruptions, partial occlusions, and intraoperative disturbances [5,6]. These limitations have been highlighted in the recent literature, motivating the need for complementary perception mechanisms capable of increasing robustness under realistic clinical conditions [7]. Markerless, vision-based perception recently emerged as a promising alternative for surgical guidance, supported by advances in deep learning-based object detection and segmentation. In medical imaging, the YOLO-based architecture has gained widespread adoption due to its favorable trade-off between accuracy and real-time performance, with recent surveys reporting competitive results across a broad range of clinical detection and segmentation tasks [8,9]. These characteristics make YOLO-family models particularly suitable for intraoperative applications, where low latency and computational efficiency are critical requirements. In parallel, foundation segmentation models such as the SAM have enabled efficient generation of high-quality segmentation masks with limited manual supervision. Recent studies have demonstrated that hybrid approaches combining YOLO-based detectors with SAM can significantly accelerate dataset annotation workflows while preserving segmentation quality, making them attractive for domain-specific medical imaging applications where large, annotated datasets are difficult to obtain [10,11]. A recent systematic review of YOLO applications in dentistry confirms the rapid adoption of YOLO-family detectors and segmenters across panoramic, CBCT, and intraoral imaging tasks [12]. In dentistry, deep-learning approaches have shown increasing potential for tooth detection, segmentation, and identification, supporting tasks such as treatment planning and image-based analysis [13]. However, most existing works focus on static image analysis or offline processing, with limited emphasis on real-time robustness under intraoperative conditions such as variable illumination, specular reflections caused by saliva, partial occlusions, and camera motion. Moreover, the integration of tooth-level perception as a dedicated verification or support layer within robotic or navigation systems remains insufficiently explored.

Beyond static image analysis, the current state of the art in medical computer vision is increasingly oriented toward real-time, instance-level perception capable of supporting decision-making and actuation under dynamic conditions. In surgical environments, segmentation is no longer regarded solely as a post-processing or diagnostic tool, but as a geometric preprocessing stage that stabilizes downstream modules such as landmark detection, spatial registration, and motion compensation. The recent literature in image-guided interventions emphasizes that perception latency, temporal consistency, and robustness to domain-specific artifacts are critical determinants of system reliability, particularly when visual outputs are directly coupled to navigation software or robotic controllers [14,15].

In medical imaging more broadly, instance segmentation has progressively replaced pure bounding-box detection in applications requiring anatomical isolation and geometric precision. Compared to detection-only approaches, instance-level masks enable more accurate boundary modeling, improved spatial correspondence, and reduced ambiguity in crowded or partially occluded scenes. This is particularly relevant in dentistry, where multiple teeth with similar morphology coexist in a confined field of view and where soft-tissue structures and reflective surfaces frequently introduce visual ambiguity. Recent reviews on deep learning in dental imaging highlight the rapid adoption of convolutional detection and segmentation frameworks across panoramic radiography, CBCT, and intraoral imaging while also noting the limited translation of these models into real-time intraoperative workflows [16].

From a systems perspective, perception modules integrated into navigation stacks must satisfy three concurrent constraints: geometric accuracy, deterministic latency, and robustness under non-ideal acquisition conditions. Studies on surgical navigation architectures report that even small inconsistencies in visual tracking or segmentation may propagate to pose estimation errors, affecting downstream spatial alignment [5]. In dentistry, intraoral imaging presents additional challenges, including specular highlights from saliva, rapid camera motion, variable illumination, and partial occlusions caused by lips or instruments. These factors necessitate perception models that degrade gracefully and maintain spatial coherence across frames rather than optimizing solely for offline segmentation accuracy.

While foundation segmentation models and hybrid detector–segmenter pipelines have demonstrated strong performance in annotation acceleration and zero-shot generalization, their multi-stage inference structure introduces potential variability in runtime and prompt sensitivity. For time-constrained navigation scenarios, particularly those targeting integration with motion compensation or robotic execution, a consolidated single-stage YOLO-seg model offers advantages in terms of a predictable computational footprint and reduced error propagation across sequential modules [17]. In this context, domain-trained YOLO-based segmentation models represent a pragmatic compromise between accuracy and real-time feasibility, enabling per-tooth mask extraction at frame rates compatible with live intraoral imaging.

Consequently, advancing markerless dental navigation requires not only improved segmentation accuracy but also a system-level perspective in which instance-level perception is explicitly designed as a stable geometric interface between visual sensing and navigation or robotic control layers. The present work addresses this gap by evaluating a real-time, single-stage tooth segmentation pipeline under both quality and latency constraints, positioning it as a 2D perception framework for future integration with stereo-based 3D reconstruction and robot-assisted implant placement.

In this paper, a YOLOv8-based tooth detection and segmentation pipeline designed as a real-time, markerless perception module for dental navigation systems is presented. Building upon previous work on navigation and robot-assisted implant placement [7], the proposed approach aims to complement existing tracking methods by providing direct visual understanding of dental anatomy from RGB images. The dataset was constructed through manual polygon labeling by the authors, with SAM employed only as a baseline segmentation pipeline for comparison. The proposed pipeline is evaluated using standard detection metrics, qualitative visual analysis, and runtime measurements to assess its feasibility for real-time deployment.

## 2. Materials and Methods

### 2.1. Real-Time Deep Learning-Based Perception System

**Figure 1:**
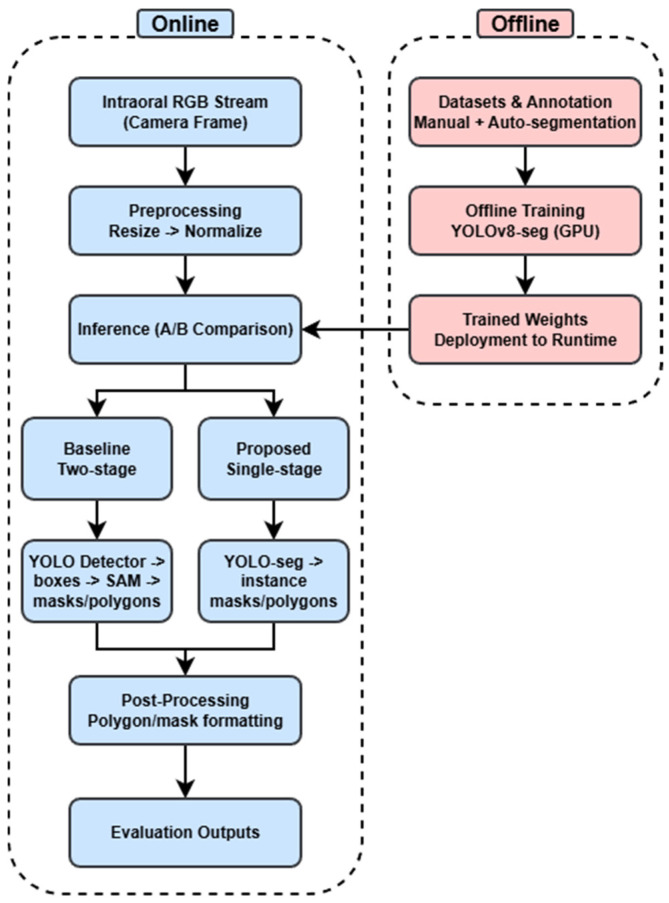
System architecture for real-time markerless tooth detection and segmentation.

The deep learning-based perception system for real-time, markerless tooth detection and segmentation in intraoral RGB images is designed as a modular pipeline that explicitly separates offline model training from online inference and deployment, ensuring reproducibility during development and deterministic behavior at runtime (see Figure 1).

During online operation, image frames acquired from an intraoral imaging device are first resized and normalized to match the network input requirements. The preprocessed images are then processed by a YOLOv8-based instance segmentation model, which performs per-tooth detection and mask prediction in real time. The network outputs bounding boxes, class labels, confidence scores, and pixel-level segmentation masks for individual teeth. A post-processing stage converts raw predictions into a standardized representation, such as polygonal masks with consistent class identifiers, and exposes them through a unified interface. This abstraction enables seamless integration of the perception module with downstream navigation or robot-assisted dental systems. All model training and optimization steps are performed offline using a dedicated dataset and GPU acceleration. Only the trained model weights are deployed in the online system, minimizing computational overhead and preserving real-time performance.

To ground the present markerless perception pipeline within an established experimental context, the present work builds upon a previously developed robotic dynamic navigation framework that initially relied on fiducial markers for tracking and guidance. In that earlier system, a collaborative robot (KUKA iiwa) was integrated with optical marker tracking and real-time pose estimation to compensate for patient motion and maintain target alignment during dental implant procedures, enabling motion-compensated navigation with clinically acceptable spatial accuracy [7]. While that method demonstrated the feasibility of motion compensation in a live procedural context, it depended on external markers that introduced workflow complexity and were sensitive to occlusions.

**Figure 2:**
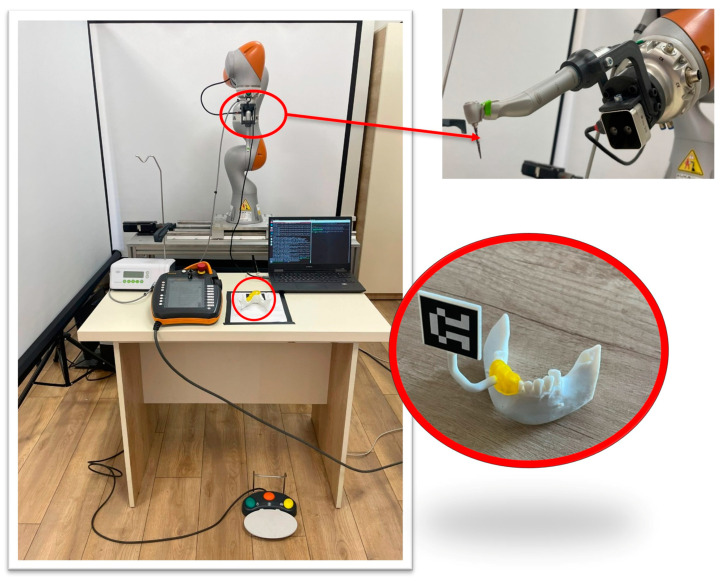
Marker-based robotic dental navigation previously reported by Pisla et al. [18].

In contrast, the current study replaces the marker-based perception stage shown in Figure 2 with a markerless, vision-based instance segmentation and detection module tailored for intraoral scenes. The goal remains the same, offering spatially consistent geometric information to downstream navigation and control layers, although the sensing modality shifts from markers to learned visual representations. This evolution supports reduced calibration steps, fewer procedural dependencies, and enhanced robustness under occlusions caused by instruments or soft tissue [18].

### 2.2. Dataset and Image Preparation

Experiments were conducted on two complementary intraoral RGB image sources combined into a single training and evaluation corpus [19].

The first source is the publicly available “Front View 3” intraoral dataset released through Roboflow Universe by the University of Bristol under a CC BY 4.0 license (200 source frontal intraoral images). Images were exported at a uniform resolution of 640 × 640 after EXIF-aware auto-orientation and aspect-stretch resizing. A photometric augmentation policy (random brightness shift in the range of ±5%) was applied at export time, producing three variants per source image. The standard Roboflow split (302 training, 20 validation, and 50 testing) was retained.

The second source is an in-house intraoral image set acquired at a partner university hospital under the approved research program referenced in the Acknowledgments Section (98 training and 50 testing images, captured between May and October 2025 with handheld intraoral devices on consenting adult subjects). These images were manually annotated by the authors using closed-contour polygons following the same nine-category taxonomy as the public dataset: lower and upper canines (C Lower/C Upper), central incisors (CI Lower/CI Upper), lateral incisors (LI Lower/LI Upper), first premolars (P1 Lower/P1 Upper), and a generic “Rear” class covering posterior teeth not individually resolvable from the frontal view. Annotations are stored in YOLO-seg format (one class index followed by normalized polygon vertices per instance), making them directly compatible with the public-dataset labels and with the polygon outputs produced by both pipelines [20].

The combined corpus therefore comprises 400 training, 20 validation and 100 testing images. The SAM vs. YOLO-seg comparison reported in Section 3.3 (Table 1) was performed exclusively on the 50 partner-hospital test images, which constitute a held-out clinical test set disjoint from all training and validation data and re-annotated to a higher standard than the public-dataset labels. No image from this evaluation set was used during training or hyperparameter selection of either pipeline. Patient-level metadata (age, gender, and dental condition) is not associated with the released annotations; this is acknowledged as a limitation in Section 4.3.

Prior to training and evaluation, images were processed to ensure a consistent input space (e.g., fixed network input size via resizing with aspect-ratio preservation and padding when required). This is particularly important for polygon-based segmentation, since non-uniform scaling can distort tooth boundaries and bias overlap metrics. Basic dataset checks were performed to confirm label availability and to ensure segmentation experiments were conducted only on polygon-annotated samples.

### 2.3. Manual Polygon Annotation Protocol

Ground-truth tooth masks were produced via manual polygon annotation, where each visible tooth instance was outlined by a sequence of vertices forming a closed contour. Annotations were stored in YOLO-seg format, with one label file per image and one line per instance. Each line contains the class index followed by normalized polygon coordinates, enabling a compact representation that can be converted deterministically into pixel-space masks for evaluation.

To ensure label consistency across the dataset, a fixed class was used throughout annotation. When teeth were only partially visible due to a limited field of view, soft-tissue coverage, or specular reflections, polygons followed the visible boundary of the crown and did not extrapolate into occluded regions. Overlapping instances were avoided by assigning each pixel to a single tooth outline, and small isolated artifacts were excluded to prevent noisy masks. These rules were applied uniformly so that both training targets and evaluation masks reflect realistic intraoral visibility.

### 2.4. SAM-Assisted Segmentation Pipeline (Baseline)

As a baseline, a two-stage segmentation workflow that combines a trained YOLO detector with the SAM was implemented [10]. In the first stage, the YOLO model predicts tooth bounding boxes for each input image. These detections are then used as prompts for SAM, which generates instance masks for the corresponding tooth regions. The resulting masks are exported as polygon annotations in YOLO-seg format, maintaining a consistent representation with the manual ground truth and the YOLO-seg outputs.

This baseline was primarily used to evaluate whether a general-purpose segmentation model, guided by detector prompts, can provide adequate tooth masks without requiring dense manual labeling for every image. Because SAM inference and polygon export introduce substantial per-frame overhead, the pipeline is benchmarked under both inference-only and end-to-end timing conditions in a matched comparison with the single-stage model described in Section 2.5.

### 2.5. Single-Stage YOLO-Segmentation Method

A single-stage YOLO-seg model [21] was adopted to perform tooth instance segmentation directly from RGB images in one forward pass. Unlike the YOLO + SAM baseline, this approach does not require an additional segmentation stage after detection, reducing pipeline complexity and making it better suited for time-constrained operation.

The model was trained on manually annotated polygon masks following the class taxonomy defined in the annotation protocol. Training followed the standard Ultralytics segmentation workflow with GPU acceleration (CUDA). A fixed input resolution was used during training to ensure consistent geometry handling for polygon-based masks, and the optimization step was run for a predefined number of epochs with a fixed batch size. Standard training augmentations provided by the framework were enabled to improve robustness to lighting variability and partial occlusions common in intraoral imaging.

The motivation for adopting YOLO-seg is methodological. It consolidates detection and segmentation into a single model, eliminating dependence on external segmentation models and reducing the number of stages that must run sequentially. This design choice supports integration into a larger navigation pipeline where per-tooth instance masks are required as stable inputs for subsequent steps such as landmark extraction and depth-based 3D mapping.

### 2.6. Runtime Benchmarking Protocol

Runtime was assessed using a pseudo-stream protocol on a sequence of N = 100 intraoral frames processed sequentially at a batch size of 1 and an input resolution of 640 × 640 on a single NVIDIA RTX A2000 (12 GB) GPU (NVIDIA Corporation, Santa Clara, CA, USA) with CUDA 12.1 and PyTorch 2.5.1. To avoid the asymmetric comparison common in detector-plus-promptable-segmenter pipelines, both methods were benchmarked under two matched conditions:

(1) **Inference-only**: Only the forward pass of the model(s) is timed; for YOLO-seg this is a single model.predict call; for YOLO + SAM this is the YOLO detection forward pass followed by a SAM forward pass on the resulting boxes, with no disk I/O.

(2) **End-to-end**: This represents inference plus serialization of the predicted polygons to a YOLO-format .txt file, reflecting practical logging or dataset-augmentation use cases. Each frame is timed independently using a high-resolution monotonic clock with explicit cuda.synchronize() calls before start and stop. A warm-up of 10 frames is run for both methods before timing to stabilize GPU kernels and CUDA caches. Reported values are the mean and standard deviation across *N* = 100 frames, with 95% bootstrap confidence intervals (10,000 resamples) on the mean.

**Figure 4:**
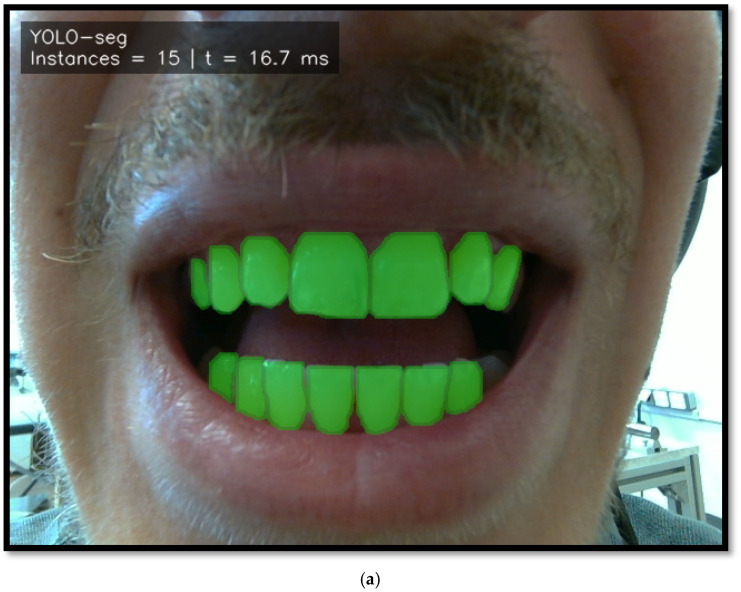

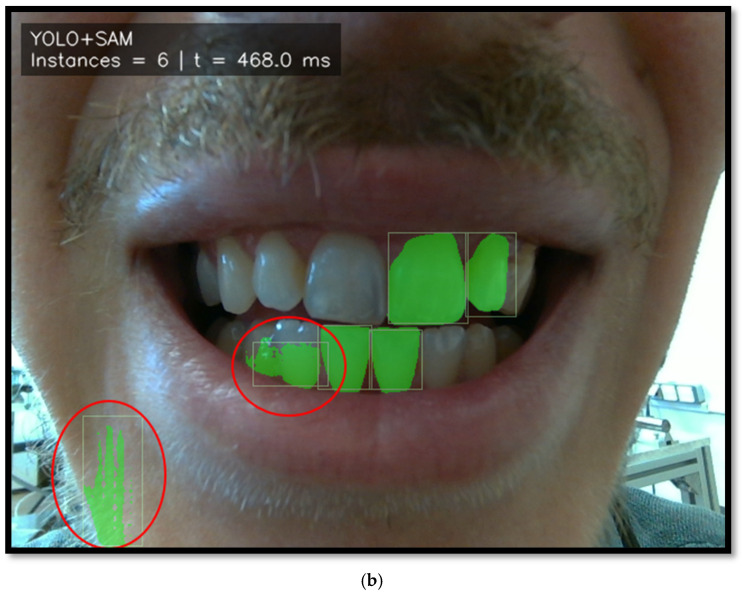
(**a**) Single-stage YOLO-segmentation overlay. (**b**) Detector-guided YOLO + SAM overlay.

In addition to the pseudo-stream benchmark, a live acquisition test was performed using an Intel RealSense camera (Intel Corporation, Santa Clara, CA, USA) streaming intraoral frames in real time. This setup includes sensor capture and visualization overhead beyond model inference and was used as a complementary qualitative check to ensure that the latency budget established under the pseudo-stream condition holds under real acquisition. The frames shown in Figure 4 originate from this live RealSense stream.

### 2.7. Segmentation Quality Evaluation

Segmentation performance was evaluated at three complementary levels, each addressing a distinct aspect of mask quality:

1. **Per-instance matching (primary).** Predicted and ground-truth instances were greedily matched within the same tooth category at a range of IoU thresholds. We report precision and recall at IoU ≥ 0.5, average precision at IoU = 0.5 (AP_50_) and the COCO-style AP_50:95_ averaged over IoU thresholds in [0.50, 0.95] in steps of 0.05. This is the level at which instance segmentation methods must be evaluated to expose failure modes such as missed teeth, duplicated detections, and mask fragmentation.
2. **Per-class union (secondary)**. Instances belonging to the same tooth category were unioned into a single binary mask per class. IoU and Dice were computed per class, and the per-image score was the mean over classes present in the ground truth. This level isolates category-level coverage from instance counting.
3. **Global union (legacy)**. All instances were unioned into a single foreground mask. This level reflects the simplest, most permissive notion of tooth-region coverage and is reported for transparency and direct comparability with prior work using this convention while explicitly acknowledging that it inflates the apparent overlap and masks per-instance failure modes.

Both ground-truth and predicted polygons were rasterized at the native image resolution using a closed-contour fill, and all metrics were computed in the same pixel space.

Instance segmentation performance was additionally assessed using the Ultralytics YOLOv8 segmentation validator on the evaluation split. We report mask precision (P), recall (R), and mean average precision (mAP) at IoU thresholds of 0.5 and 0.5:0.95 (COCO-style), together with the corresponding box metrics for completeness. A low confidence threshold (conf = 0.001) was used during validation to enable proper precision–recall curve estimation.

#### Statistical Analysis

Quality metrics are paired by image across the two methods. We therefore use the Wilcoxon signed-rank test (one-sided, YOLO-seg > YOLO + SAM) for hypothesis testing [22], which does not assume normality of the per-image score distributions. Effect size is reported as Cliff’s delta [23], a non-parametric ordinal effect size with a range of [−1, 1]. Uncertainty on per-method means and on the YOLO-seg minus YOLO + SAM difference is reported as 95% percentile bootstrap confidence intervals (10,000 resamples) on the corresponding statistic. Statistical significance is set at α = 0.05.

### 2.8. Inference Procedure and Output Standardization

For both pipelines, predictions were filtered with standard non-maximum suppression and exported in a unified polygon format aligned with the dataset annotation scheme. Each predicted instance was represented by a class label, a confidence score, and a polygon defined by normalized vertex coordinates, enabling direct comparison with the manual ground truth in the same pixel space.

This standardized representation was used as the common interface for evaluation and for subsequent processing stages in the proposed navigation-oriented workflow. The experiments were executed on a Windows 11 Pro equipped with an NVIDIA RTX A2000 12 GB GPU (NVidia Corporation, Santa Clara, CA, USA), an Intel 12th Gen Core i9-12900K CPU (NVidia Corporation, Santa Clara, CA, USA) and 32 GB of DDR5 RAM (NVidia Corporation, Santa Clara, CA, USA).

## 3. Results

### 3.1. Annotation Pipeline and Representation Formats

Before reporting quantitative benchmarks, we illustrate how manual polygon annotations are converted into the binary masks and bounding boxes used as the ground truth for evaluation. This visualization confirms that the same polygon labels can be deterministically expressed in all three representations needed by the two pipelines.

**Figure 3:**
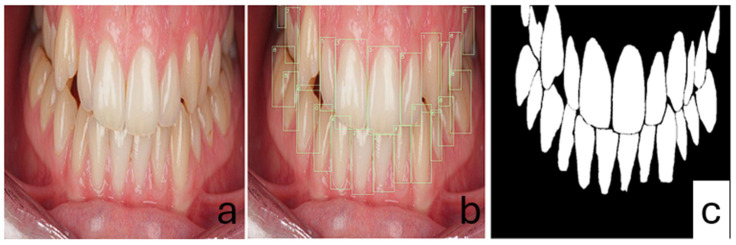
(**a**) Original image, (**b**) bounding Boxes, and (**c**) binary Mask.

Figure 3a–c illustrate the qualitative stages of the proposed tooth perception workflow, from raw intraoral image input to structured geometric representations. Starting from the original RGB image, manually annotated polygon labels are rasterized into binary masks, providing pixel-level ground-truth representations of visible tooth regions. From these same polygon annotations, corresponding bounding boxes are deterministically derived by computing the spatial extrema of each instance. This visualization confirms that the adopted annotation format allows consistent conversion between polygonal, mask-based, and bounding-box representations, enabling a direct comparison across detection- and segmentation-based pipelines without additional geometric approximations.

### 3.2. Qualitative Comparison Between YOLO-Seg and YOLO + SAM

Figure 4 compares the qualitative behavior of YOLO-seg and the detector-guided YOLO + SAM baseline on a representative intraoral frame acquired live with an Intel RealSense camera. In the YOLO + SAM pipeline, tooth regions are first localized by the detector and then used as box prompts for SAM, so mask quality depends on both detection and prompt-guided segmentation. In contrast, YOLO-seg predicts instance masks directly in a single forward pass, eliminating second-stage dependency.

In the evaluated examples, YOLO-seg (Figure 4a) produces more spatially coherent tooth masks with smoother, more compact regions and limited spill into the surrounding soft tissues. The YOLO + SAM output (Figure 4b) generally captures the coarse extent of teeth but shows prompt-dependent artifacts such as fragmented regions, boundary irregularities, and occasional leakage into non-tooth structures when strong edges are present (e.g., lips, facial hair, or specular highlights). This qualitative difference is consistent with a two-stage approach in which imperfect bounding boxes propagate to segmentation, especially under intraoral lighting variability. Overall, Figure 4 supports the use of YOLO-seg as the primary perception module for time-constrained operation, while the YOLO + SAM baseline retains value primarily as an annotation-acceleration tool rather than as an online perception module.

### 3.3. Segmentation Quality Evaluation

Segmentation performance was quantified against the manually annotated polygon ground truth on N = 50 images, with predicted and ground-truth polygons rasterized at the native image resolution. Results are summarized in Table 1 at three evaluation levels (per-instance, per-class union, and legacy global union). The single-stage YOLO-seg pipeline consistently and significantly outperformed the YOLO + SAM baseline across all primary metrics, with very large effect sizes (Cliff’s delta in 0.76–0.94 across instance-level and per-class metrics) and Wilcoxon *p*-values below 1 × 10^−8^ on every metric except precision at IoU = 0.5 (*p* = 9.67 × 10^−3^, delta = 0.29). The gap is particularly visible at the per-instance level: YOLO + SAM only achieved AP_50_ = 0.383 (95% CI [0.307, 0.460]) and recall = 0.398 (95% CI [0.318, 0.481]) at IoU ≥ 0.5, indicating systematic instance-level failures despite acceptable global coverage. YOLO-seg achieved AP_50_ = 0.716 (95% CI [0.682, 0.738]) and recall = 0.973 (95% CI [0.928, 0.999]), indicating that on this held-out test set the single-stage model recovered the correct number and identity of teeth in the large majority of frames. Given the limited size of this set (N = 50), these values should be interpreted as evidence on the present clinical sample rather than as a guarantee of generalization to broader populations. The discrepancy between the legacy global-union IoU (gap = 0.236) and the per-class IoU (gap = 0.586) directly illustrates why a single foreground mask is inadequate to characterize instance segmentation quality.

While the three-level evaluation in Table 1 is based on paired polygon rasterization against the partner-hospital annotations, we additionally report the output of the Ultralytics YOLOv8 segmentation validator on the same evaluation split as a standardized instance-level reference (Table 2). The validator uses its own matching protocol (COCO-style IoU sweep with greedy assignment) and reports mask *p* = 0.870, mask R = 0.890, mask mAP@0.5 = 0.907 and mask mAP@0.5:0.95 = 0.695. The corresponding box metrics were *p* = 0.875, R = 0.895, mAP@0.5 = 0.915, and mAP@0.5:0.95 = 0.775 (Table 2). These figures are consistent with the per-instance results in Table 1 and confirm the single-stage model’s strong instance-level performance under the COCO evaluation protocol.

### 3.4. Runtime Feasibility

To assess whether the tested pipelines can operate under time constraints relevant to live intraoral imaging, end-to-end runtime was measured on N = 100 frames using the pseudo-stream protocol described in Section 2.6. To eliminate comparison bias, both methods were timed under matched conditions: an inference-only mode (model forward pass only) and an end-to-end mode (inference plus polygon serialization). Results are summarized in Table 3.

Under the inference-only condition, YOLO-seg ran at 27.08 ± 12.12 ms per frame (median: 25.24 ms; 95% CI: [24.86, 29.56] ms; 36.9 FPS), whereas YOLO + SAM required 1302.78 ± 151.54 ms (median: 1328.18 ms; 95% CI: [1271.18, 1330.96] ms; 0.77 FPS)—a ~48-fold latency gap intrinsic to the two-stage forward pass and not attributable to I/O overhead. Adding the polygon-export step shifted YOLO-seg to 37.94 ± 11.51 ms (26.4 FPS) and YOLO + SAM to 1408.87 ± 216.73 ms (0.71 FPS); the end-to-end vs. inference-only overhead was statistically significant for both (Wilcoxon one-sided: YOLO-seg *p* = 8.16 × 10^−10^ and mean overhead: 10.86 ms; YOLO + SAM *p* = 2.46 × 10^−15^ and mean overhead: 106.09 ms). These figures confirm that YOLO-seg meets the latency budget for live intraoral imaging (≥25 FPS, achieved in both modes) while YOLO + SAM operates an order of magnitude below interactive frame rates regardless of whether logging is enabled.

Both pipelines were implemented in PyTorch 2.5.1 using the Ultralytics interface for YOLO-seg and the SAM interface for YOLO + SAM; both scripts include an explicit warm-up phase to reduce first-frame GPU overhead.

For a navigation-oriented system, exporting polygons to a disk is not part of the online loop. In the final architecture, predictions would instead be passed in memory as mask arrays or polygons into downstream geometry modules, and they are only optionally logged for debugging or dataset growth.

## 4. Discussion

### 4.1. Key Findings and Interpretation

The main outcome of this study is that a single-stage YOLO-seg model can provide both higher agreement with manually annotated tooth regions and substantially lower inference latency compared to a detector-guided YOLO + SAM baseline under the tested intraoral conditions. Notably, the gap between mAP@0.5 and mAP@0.5:0.95 indicates sensitivity to stricter boundary alignment requirements, which is consistent with intraoral artifacts such as partial occlusions and reinforces the emphasis on stable instance masks for their use in navigation-oriented pipelines. The segmentation quality results indicate that direct end-to-end training on polygon-derived tooth masks yields more consistent coverage of visible crowns, while the runtime benchmark highlights that eliminating the second-stage segmentation step is decisive for time-constrained operation. Together, these findings support the selection of single-stage segmentation as the primary perception layer when the objective is stable, with frequent updates of tooth regions rather than offline mask generation.

The qualitative comparison further clarifies why these quantitative differences matter in practice. In challenging live frames containing specular reflections, soft-tissue boundaries, and partial occlusions, the YOLO + SAM pipeline can exhibit prompt-dependent artifacts such as mask fragmentation and leakage into non-tooth structures. This behavior reflects its dependence on detection prompts and downstream segmentation quality, an issue also reported in recent work on real-time instrument segmentation for robotic surgery, where inference speed and segmentation stability are critical for intraoperative use [24]. In contrast, YOLO-seg tends to produce more spatially coherent masks that degrade more gracefully when imaging conditions deteriorate. For navigation-oriented use, this stability is important because downstream modules typically require consistent geometric inputs (tooth regions used for tracking, landmark extraction, or later 3D fusion) rather than intermittent high-quality segmentation on isolated frames.

### 4.2. Implications for Markerless Dynamic Navigation and Robotic Automation

This work should be interpreted as an initial step toward a markerless, vision-driven dental navigation workflow rather than a complete navigation system. Recent evidence syntheses show that computer-assisted implant surgery, dynamic navigation and robot-assisted approaches can improve placement accuracy compared with freehand workflows while also highlighting that real-world performance depends strongly on system architecture, calibration, and intraoperative constraints. Reliable segmentation supports consistent isolation of each tooth region, which is essential for robust landmark detection (e.g., cusp tips and other stable anatomical points). These landmarks can then be matched across stereo views to enable 3D reconstruction and spatial transposition from 2D image space into a navigation software environment, achieving accurate geometric models in complex imaging scenarios [25]. Within this architecture, segmentation is not an end goal; it is the gating step that stabilizes downstream landmark extraction and reduces ambiguity introduced by soft-tissue occlusions and specular artifacts typical of intraoral scenes.

From an automation perspective, the results suggest that a single-stage segmentation model is a reasonable candidate for the perception layer of a future robot-assisted implant placement system, although this remains a hypothesis to be tested rather than a demonstrated capability. A practical robotic workflow requires frequent and stable updates of the visible tooth geometry so that downstream estimation remains temporally consistent. In the envisioned integration, the reconstructed 3D dental model and extracted landmark set would be used to compute and continuously verify the target implant pose within a dedicated software pipeline, which would then communicate motion objectives to the robot controller. The robot-mounted physiodispenser and the implant placement sequence operate under strict safety constraints. Perception latency and segmentation stability are therefore directly relevant, since segmentation failure modes such as mask leakage or fragmentation can propagate to landmark errors and, in turn, to incorrect geometric correspondence in 3D. Therefore, the combination of significantly higher per-instance segmentation quality (AP_50_ = 0.716 vs. 0.383; *p* = 5.18 × 10^−9^; Cliff’s delta = 0.76) and approximately 48-fold lower inference-only latency observed for the single-stage YOLO-seg approach establishes it as a viable 2D perception front-end. Closed-loop validation against 3D registration accuracy, implant-placement error, and robotic execution under realistic intraoral motion remains future work and is not claimed here.

### 4.3. Limitations

This study focuses on validating the 2D perception layer (tooth detection and instance segmentation) as a prerequisite for a markerless navigation workflow, rather than demonstrating a complete navigation system end to end. As a result, stereo correspondence, 3D reconstruction, landmark-based pose estimation, and robot execution are intentionally left outside the current experimental scope.

The runtime evaluation reported here uses a pseudo-stream protocol in which frames previously captured on the dataset are processed sequentially as if they originated from a live sensor. This deliberately removes the variability introduced by sensor exposure changes, autofocus, motion blur, rolling-shutter artifacts, and intraoperative occlusions caused by lips, the tongue, instruments and aspirators. As a consequence, the latency figures reported here represent a lower bound on what an end-to-end intraoperative deployment would achieve, and the segmentation-quality figures represent an upper bound on what would be observed under real intraoral dynamics. A targeted in vivo evaluation under realistic motion and lighting conditions is required before any clinical claim can be made and is part of the planned next step of this work. In addition, the N = 50 evaluation set is sufficient to establish the very large effect sizes reported here (Cliff’s delta ≥ 0.76 on all primary metrics, with paired Wilcoxon *p* < 1 × 10^−8^). However, it is not large enough to characterize tail behavior on rare anatomical or imaging configurations [26]. This reflects a well-documented constraint in medical AI evaluation, where dataset size trades off against generalizability; expansion of the evaluation set with cross-validation over multiple acquisition sources is planned for the next iteration of this work.

## 5. Conclusions

In this study, we developed and evaluated a real-time tooth detection and instance segmentation pipeline based on YOLOv8, which was designed to function as a markerless perception component within dental navigation systems. The results indicate that a single-stage YOLO-seg model can achieve a stable balance between geometric accuracy and computational efficiency, making it a viable candidate for the 2D perception layer of future markerless dental navigation systems, where predictable latency is essential.

Rather than treating segmentation as an isolated image processing task, the proposed approach considers it a structural layer within a broader navigation architecture. By extracting per-tooth masks directly from RGB images, the system provides spatially consistent visual information that may support downstream processes such as landmark localization, pose estimation, or motion compensation. This perspective shifts the focus from purely maximizing segmentation scores toward ensuring stability and integration readiness within real clinical workflows.

At the same time, the current study remains limited to 2D intraoral imagery and does not include volumetric registration or closed-loop robotic validation. Performance under more challenging clinical conditions, including extreme occlusion or lighting variability, warrants further investigation into expanded datasets.

Future work will concentrate on integrating the proposed markerless perception module into the previously developed motion-compensated robotic framework, as well as exploring CBCT-based volumetric alignment to enable multimodal 3D integration. These developments aim to move progressively toward a fully vision-guided, marker-independent dental navigation system.

## Figures and Tables

**Table 1:** Segmentation quality on the partner-hospital held-out clinical test set (N = 50 images, manually re-annotated; paired across methods). Values are the mean ± SD; 95% CIs from percentile bootstrap (10,000 resamples); *p* from the one-sided Wilcoxon signed-rank test (YOLO-seg > YOLO + SAM); effect size is Cliff’s delta.

Level	Metric	YOLO + SAM Mean ± SD [95% CI]	YOLO-Seg Mean ± SD [95% CI]	Diff [95% CI]	*p* (Wilcoxon)	Cliff’s δ
Per-instance	AP @ 0.50	0.383 ± 0.275 [0.307, 0.460]	0.716 ± 0.110 [0.682, 0.738]	0.333 [0.260, 0.406]	5.18 × 10^−9^	0.757
Per-instance	AP @ 0.50:0.95	0.328 ± 0.228 [0.266, 0.390]	0.611 ± 0.102 [0.580, 0.634]	0.284 [0.223, 0.345]	1.03 × 10^−9^	0.802
Per-instance	Precision @ IoU 0.5	0.368 ± 0.287 [0.293, 0.449]	0.459 ± 0.098 [0.429, 0.482]	0.091 [0.015, 0.165]	9.67 × 10^−3^	0.292
Per-instance	Recall @ IoU 0.5	0.398 ± 0.300 [0.318, 0.481]	0.973 ± 0.143 [0.928, 0.999]	0.575 [0.490, 0.660]	7.96 × 10^−10^	0.936
Per-class	IoU (mean over c)	0.241 ± 0.196 [0.188, 0.295]	0.827 ± 0.182 [0.770, 0.870]	0.586 [0.520, 0.649]	8.88 × 10^−16^	0.936
Per-class	Dice (mean over c)	0.287 ± 0.219 [0.228, 0.347]	0.870 ± 0.189 [0.812, 0.915]	0.583 [0.513, 0.650]	8.88 × 10^−16^	0.934
Global union	IoU (legacy)	0.634 ± 0.187 [0.580, 0.682]	0.870 ± 0.178 [0.815, 0.909]	0.236 [0.199, 0.273]	1.78 × 10^−15^	0.901
Global union	Dice (legacy)	0.755 ± 0.189 [0.699, 0.802]	0.913 ± 0.181 [0.857, 0.952]	0.159 [0.131, 0.190]	1.78 × 10^−15^	0.901

**Table 2:** Instance segmentation performance of the YOLO-seg pipeline (YOLOv8 validator).

Metric	Box	Mask
Precision (P)	0.875	0.870
Recall (R)	0.895	0.890
mAP@0.5	0.915	0.907
mAP@0.5:0.95	0.775	0.695

**Table 3:** Per-frame runtime on N = 100 intraoral images, with batch: 1, imgsz = 640, conf = 0.25, RTX A2000 12 GB, CUDA 12.1, and PyTorch 2.5.1. The mean ± SD and median are reported in ms, with the 95% CI on the mean from 10,000-resample percentile bootstrap.

Method	Mode	N	Mean ± SD (ms)	Median (ms)	95% CI Mean (ms)	FPS
YOLO-seg	Inference-only	100	27.08 ± 12.12	25.24	[24.86, 29.56]	36.93
YOLO-seg	End-to-end	100	37.94 ± 11.51	38.77	[35.92, 40.36]	26.36
YOLO + SAM	Inference-only	100	1302.78 ± 151.54	1328.18	[1271.18, 1330.96]	0.77
YOLO + SAM	End-to-end	100	1408.87 ± 216.73	1420.15	[1365.88, 1450.18]	0.71

## Data Availability

The data supporting the results reported in this study are not publicly available. Part of the data were collected from clinical cases and their use was approved by the Ethics Committee; public disclosure is therefore restricted due to privacy and ethical considerations. The remaining data may be made available upon reasonable request to the corresponding author.
